# Studies on genome size estimation, chromosome number, gametophyte development and plant morphology of salt-tolerant halophyte *Suaeda salsa*

**DOI:** 10.1186/s12870-019-2080-8

**Published:** 2019-11-06

**Authors:** Yan Cheng, Pan Yang, Lihua Zhao, S. V. G. N. Priyadarshani, Qiao Zhou, Zeyun Li, Weimin Li, Junjie Xiong, Zhibin Lin, Li Li, Xinyu Huang, Jindian Liu, Mohammad Aslam, Heming Zhao, Gang Li, Jinbiao Ma, Lei Li, Yuan Qin

**Affiliations:** 1 State Key Laboratory of Ecological Pest Control for Fujian and Taiwan Crops, College of Plant Protection, Fujian Provincial Key Laboratory of Haixia Applied Plant Systems Biology, Center for Genomics and Biotechnology, Fujian Agriculture and Forestry University, Fuzhou, 350002 China; 2 College of Life Science, Fujian Agriculture and Forestry University, Fuzhou, 350002 China; 3 State Key Laboratory for Conservation and Utilization of Subtropical Agro-Bioresources, Guangxi Key Lab of Sugarcane Biology, College of Agriculture, Guangxi University, Nanning, 530004 China; 4 College of Resources and Environment, Fujian Agriculture and Forestry University, Fuzhou, 350002 China; 5 College of Crop Science, Fujian Agriculture and Forestry University, Fuzhou, 350002 China; 6 Key Laboratory of Biogeography and Bioresources in Arid Land, Xinjiang Institute of Ecology and Geography, Urumqi, 83000 China; 7 State Key Laboratory of Protein and Plant Gene Research, Peking-Tsinghua Center for Life Sciences, School of Life Sciences and School of Advanced Agricultural Sciences, Peking University, Beijing, 100871 China

**Keywords:** Soil salinization, *Suaeda salsa*, Gametophyte, Chromosome, Genome

## Abstract

**Background:**

Soil salinization and alkalization are among the major agricultural threats that affect crop productivity worldwide, which are increasing day by day with an alarming rate. In recent years, several halophytes have been investigated for their utilization in soil remediation and to decipher the mechanism of salt-tolerance in these high salt tolerant genetic repositories. *Suaeda salsa* is an annual halophytic herb in the family *Amaranthaceae*, displaying high salt and alkali-resistance and having nutritive value. However, the fundamental biological characteristics of this valuable plant remain to be elucidated until today.

**Results:**

In this study, we observed the morphology and development of *Suaeda salsa*, including seed morphology, seed germination, plant morphology, and flower development. Using microscopy, we observed the male and female gametophyte developments of *Suaeda salsa*. Also, chromosome behaviour during the meiosis of male gametophyte was studied. Eventually, the genome size of *Suaeda salsa* was estimated through flow cytometry using Arabidopsis as reference.

**Conclusions:**

Our findings suggest that the male and female gametophyte developments of *Suaeda salsa* are similar to those of the model plant Arabidopsis, and the diploid *Suaeda salsa* contains nine pairs of chromosomes. The findings also indicate that the haploid genome of *Suaeda salsa* is approximately 437.5 MB. The observations and results discussed in this study will provide an insight into future research on *Suaeda salsa*.

## Background

Soil salinization and alkalization have adverse effects on agricultural land, leading to reduced soil fertility. In recent years, salinization and alkalization have emerged as a severe threat affecting crop production worldwide [1]. It is estimated that about 20% of the world’s cultivated land and nearly half of all irrigated land are affected by salinity and alkalinity [2]. The saline-alkaline land is widely distributed in the regions of all major continents, mainly in Eurasia, Africa and the western part of the Americas [3]. China’s cultivatable land is also severely threated by the salinization and alkalization. The total area of saline-alkaline land in China is about 3.6 × 10^7^ ha, accounting for 4.88% of the available land base [4]. The matter is becoming more severe in northeast China, where the saline-alkaline meadow covers more than 70% of the land area [5]. All in all, the severity in the world is still expanding due to human activities and global climate changes [6].

*Suaeda salsa* (accepted name: *Suaeda maritima subsp. salsa (L.) Soó*) is a well-known salt and alkali-resistant, succulent halophyte in the family *Amaranthaceae,* which was first recorded in an ancient Chinese book “Jiu Huang Ben Cao” that enrolled the potential food plants to cope with famine during Ming dynasty. *Suaeda salsa* exhibits a high tolerance to salt and alkali stresses and grows very well under salt content more than 0.48% even without salt glands and bladders in its leaves [7]. The most suitable NaCl concentration for promoting its growth is 200 mM, and there is no significant difference can be observed when it is treated with 400 mM NaCl and 10 mM NaCl [8]. As a model salt-tolerant plant, a number of genes involved in salt tolerance such as *SsHKT1, SsNHX1, SsCAX1* have been identified, and their functions analyzed [9–11]. Additionally, *Suaeda salsa* possesses good Cd, Pb and Mn tolerance and could be considered as a hyperaccumulator for those heavy metals, reflecting its ecological value on recuperating heavy metals-contaminated soil [12]. In addition to the values mentioned above, *Suaeda salsa* has very high edible and medicinal values as well. It is an annual herb, with excellent palatability for domestic animals and has great value in Chinese traditional medicine [13]. The young leaves and stems of *Suaeda salsa* are a highly nutritious vegetable that contains abundant proteins, dietary fibre, vitamins, minerals, and flavonoids [14], The oil from *Suaeda salsa* seeds is also edible [15], and it is rich in fatty acids. 90.7% of *Suaeda salsa* fatty acid is unsaturated. Furthermore, the relative content of unsaturated fatty acids is higher than the other cooking oils, among which, the terephthalic acid, 11-Hexadecenoic acid, and Linoleic acid from *Suaeda salsa* seeds are up to 0.82, 0.45, 68.74% respectively [16]. It has been documented that the seed oil of *Suaeda salsa* has the function of decreasing blood sugar and blood pressure, lowering blood cholesterol, developing disease immunity [17], Therefore, the oil produced from *Suaeda salsa* seeds is beneficial for human consumption [18]. In this case, biological researchers have been putting the focus on increasing its seed yield [19].

Considering the scientific and edible values of *Suaeda salsa*, a number of researches recently have been conducted in the scopes of understanding the salt-tolerance mechanism, medicinal use, and nutrient value [1, 14, 20]. However, the reports regarding the fundamental biological characteristics of *Suaeda salsa* are limited and not systematic. In this study, the plant and flower morphologies of *Suaeda salsa* were observed, and the developments of its female and male gametophytes were described. Furthermore, the genomic characteristics of *Suaeda salsa* concerning chromosome number and genome size were also investigated. These results will improve our understanding of *Suaeda salsa* for future research and its utilization for crop breeding programme.

## Results

### Seed morphology and germination of *Suaeda salsa*

**Fig. 1:**
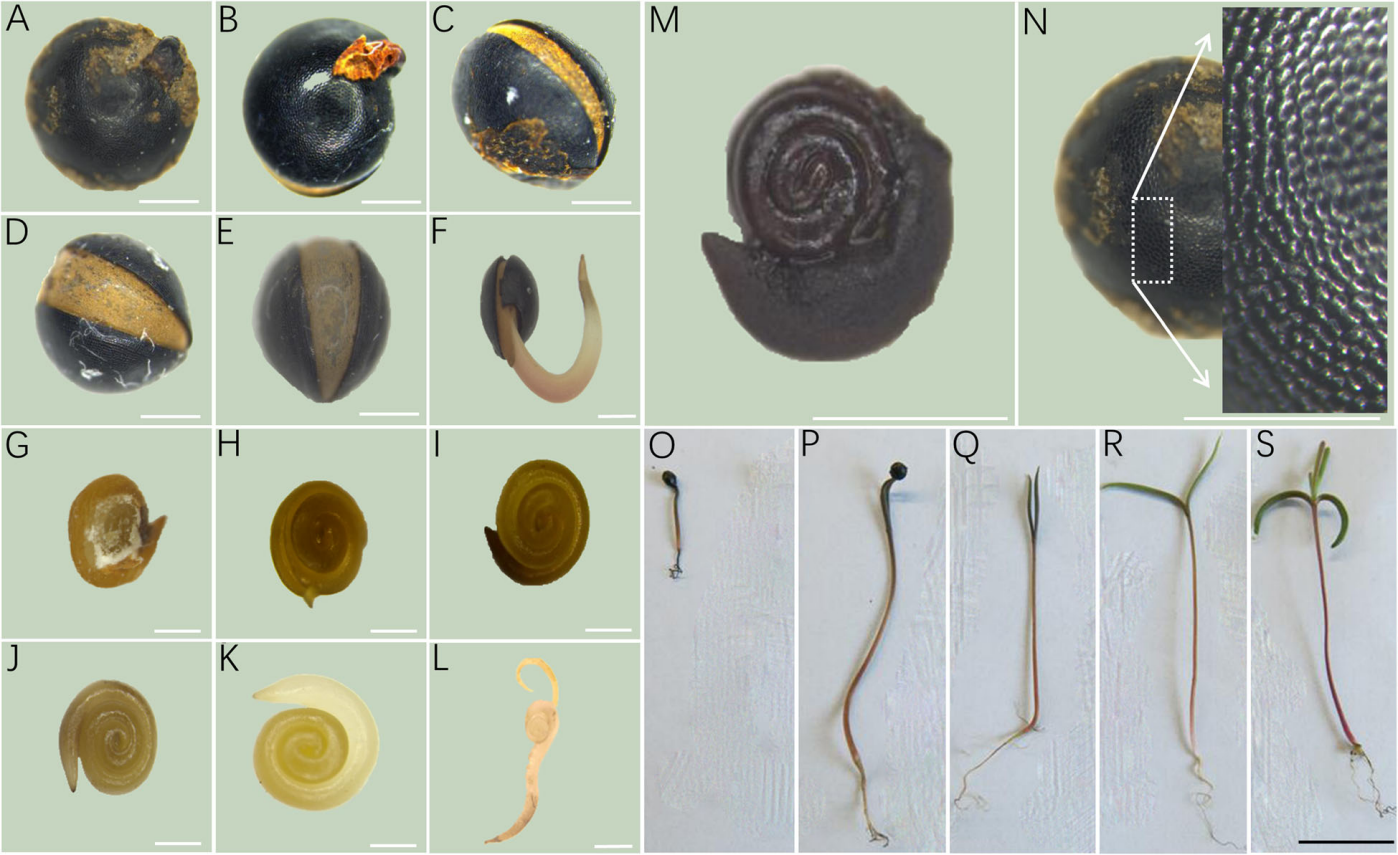
Seed structure and seed germination pattern of *Suaeda salsa*. **a-f** Endocarp appearance and early germination pattern of *Suaeda salsa*. Bar = 0.5 mm. **g-l** Seed germination pattern at different stages of planospiral embryo. Bar = 0.5 mm. **m** The Seed treated with KI/I_2_ for the starch test. Bar = 0.5 mm. **n** Endocarp having honeycomb-like structure. Bar = 0.5 mm. **o-s** Epigeal germination pattern of *Suaeda salsa*. Figures showed the seedlings at 48, 60, 72 h, and 5 and 40 days after germination. Bar = 1 cm

In angiosperms, a seed is covered with pericarp, which consists of endocarp, mesocarp, and exocarp. The germination process of *Suaeda salsa* seeds has been simply observed in a recent report, in which the roles of gibberellins and abscisic acid in regulating the germination of *Suaeda salsa* under salt stress were revealed [21]. Here, we conducted an extensive observation of the germination process of *Suaeda salsa* seeds. The mature seeds of *Suaeda salsa* also consisted of thin fleshy mesocarp and exocarp. Endocarp is hard and thin with blackish colour. During germination, endocarp was split into two parts. This splitting can be easily observed after 24 h of germination and split becoming wider after 48 h of germination, allowing radical to grow easily (Fig. 1a-f). Careful observation of the endocarp surface showed the honeycomb-like pattern (Fig. 1n). Having thin hard endocarp makes seed germination obstructed delaying the propagation process, which is required to meet the agricultural demand. When the pericarp was removed, seeds appeared flat, disc-shaped with a size of 1.8–2.1 mm in diameter. The seed has a thin brownish seed coat (Fig. 1g). Once the seed coat was removed, we observed a brown thin whitish colour layer consisted of starch, which turned to a blackish-blue colour when treated with KI/I_2_ (Fig. 1m)_._ With the start of germination, the seed coat and thin starchy layer started to disappear. At this stage, we observed mature germinating planospiral embryo that is the distinguishing feature in this plant family [22] (Fig. 1g-l). The outermost end of the planospiral embryo act as radical, giving rise to root. The innermost end of the embryo act as plumule that later develops into the shoot. During *Suaeda salsa* seeds germination, we observed radical growth into roots first and then followed by two cotyledons appearance at the other end of the embryo. Later we observed the emergence of true leaves. This germination study revealed that the *Suaeda salsa* possesses epigeal germination pattern (Fig. 1o-s).

### Plant development and morphology of *Suaeda salsa*

**Fig. 2:**
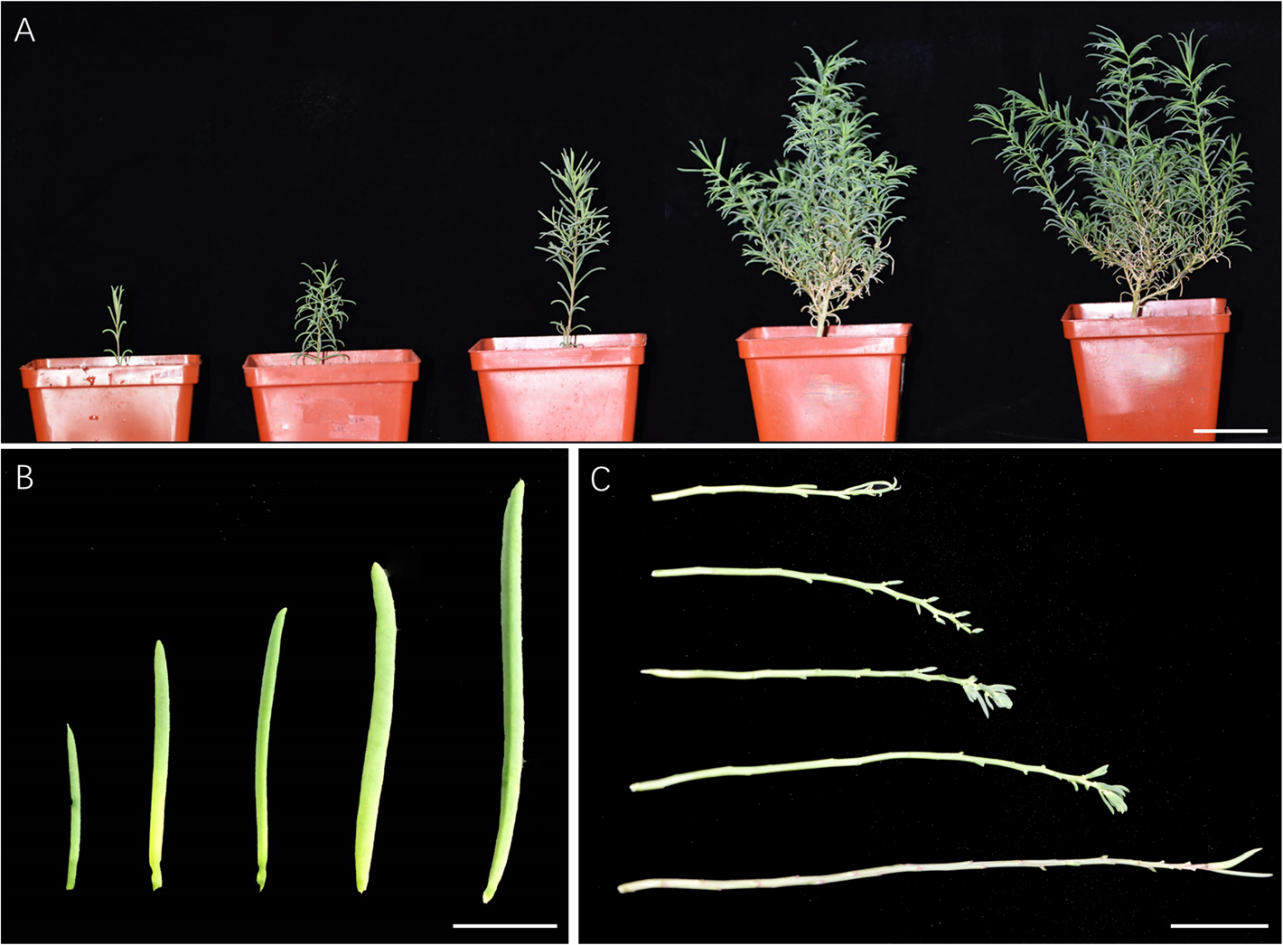
Leaves, stems, and plants of *Suaeda salsa* at five developmental stages. **a** The whole plant of 10-, 20-, 40-, 80-, and 100-days old *Suaeda salsa* grown in the greenhouse (Bar = 5 cm). **b** The 5 leaves of 100-days old *Suaeda salsa* (Bar = 2 cm). **c** The stems of 100-days old *Suaeda salsa* grown in the greenhouse (bar = 3 cm)

To understand the morphology of *Suaeda salsa,* we observed the plant architecture at five vegetative development stages. Fig 2 shows the whole plant (Fig. 2a) of 10-, 20-, 40-, 80-, and 100-days old *Suaeda salsa* grown in the greenhouse. *Suaeda salsa* plant has one main axis with branches of second-order (e.g. primary, secondary, tertiary branches). The leaves are flat to round cylindrical without recognizable petioles, and the branches emerge from 20 to 40 days without significant difference to the main stem. The leaves (Fig. 2b) and stems (Fig. 2c) from five different positions on one plant are displayed as well.

**Fig. 3:**
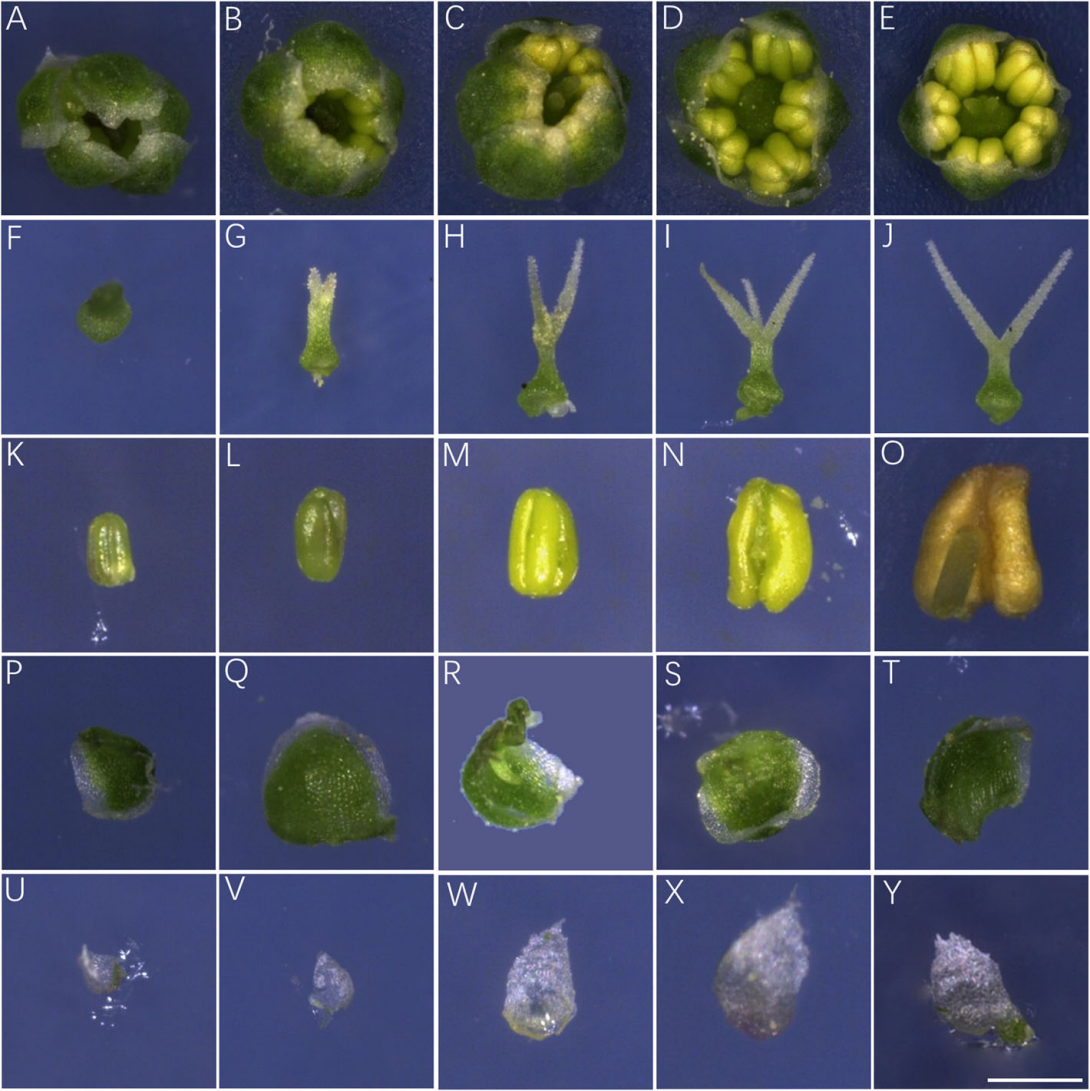
The floral organs of *Suaeda salsa* at five developmental stages. The figure showed the flower buds (**a-e**)**,** pistils (**f-j**), stamens (**k-o**), petals **(p-t)** and calyxes (**u-y**) at five developmental stages. Bars = 500 μm

The mature flowers of *Suaeda salsa* are spherical or nearly spherical, with a diameter of 2 ± 0.2 mm (Fig. 3e). They usually emerge directly on the main and lateral stems. Most of *Suaeda salsa* flowers are hermaphroditic, sometimes female flowers could be observed. Hermaphroditic *Suaeda salsa* flowers are composed of four whorls: the inner first whorl possesses gynoecium containing only one pistil with two to three stigmas; the second whorl has five stamens, each stamen possesses two pollen sacs; the third whorl contains five green petals. Interestingly, the petals are similar to the canonical calyxes because of the existence of chlorophyll. Even though five calyxes could be observed in the outer whorl, they are transparent and significantly degraded.

To accurately describe the flower development of *Suaeda salsa*, we observed the inflorescence stages of *Suaeda salsa*. According to its flower bud development characteristics, the inflorescence development was divided into five stages. Stage I (100–110 DAG): the flower buds originate from the main stems and branches, and the stamens are invisible (Fig. 3a). Stage II (110–130 DAG): the flower buds grow rapidly, and reach a maximum size, the petals were closely connected, not cracked, and the stigmas were lower than the stamens (Fig. 3b). Stage III (130–140 DAG): the petals begin to split; 1 or 2 stamens are visible. The lengths of stigmas and stamens are the same (Fig. 3c). Stage IV (140–145 DAG): the petals are completely cracked; 5 stamens are naked and light green (Fig. 3d). Stage V (145–155 DAG): the stamens mature, the colour is from light green to yellow, the stigmas are longer than the stamens (Fig. 3e).

We further dissected the flower buds from these five stages and measured the size of the flower organs. As shown in Fig. 3, A-E are the flower buds at five developmental periods, F-J are ovaries at five developmental periods, K-O are stamens at five developmental periods, P-T are petals at five developmental periods. U-Y are calyxes at five developmental periods. The quantification data of different floral parts during these five developmental stages were shown in Additional file 1: Table S1.

### Male gametophyte development of *Suaeda salsa*

**Fig. 4:**
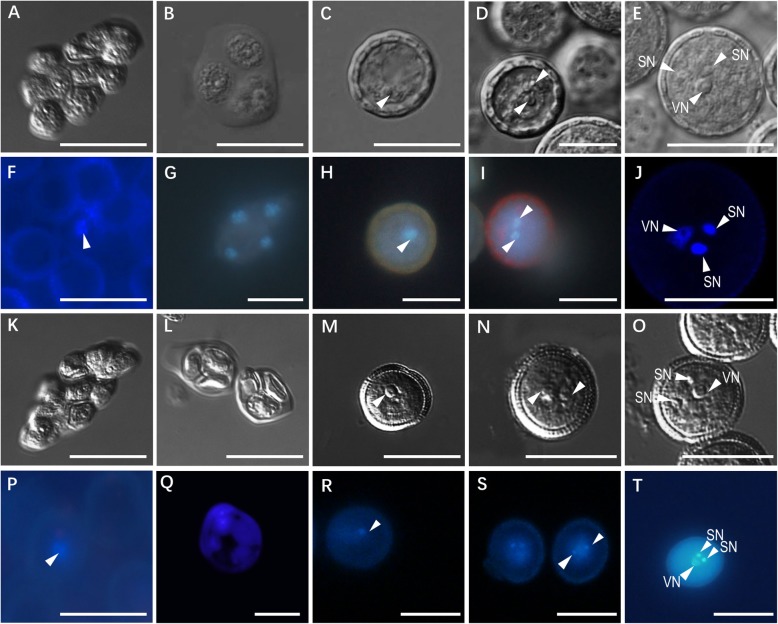
The male gametophyte development of *Suaeda salsa* (**a-j**) and Arabidopsis (**k-t**). The pollen development observations under the DIC microscope **(a-e, k-o)** and fluorescence microscope with DAPI staining **(f-j, p-t).** For each observation, five development stages are shown: pollen mother cell stage **(a, f,** k and **p)**, four haploid microspores stage (**b, g, l** and **q**), uninucleate microspore stage (**c, h, m** and **r**), binuclear pollen stage (**d**, **i**, **n** and **s)** and tricellular pollen stage (**e, j,o and t**) are shown respectively. SN, sperm nucleus, VN, vegetative nucleus. Bar = 20 μm

Gametogenesis is a fundamental and critical step in plant life cycles [23], which contributes to the formation of the male gametophyte (pollen) in male organ anther and female gametophyte (embryo sac) in female organ ovule. Although the morphology of mature pollen of *Suaeda salsa*, including pollen diameter, pore number, pore diameter, etc., have been investigated and reported [24], its male gametophyte development process remains undescribed. To this end, we observed the pollen development of *Suaeda salsa,* which was compared to that of the model plant Arabidopsis subsequently. The stamens of 5 stages (stage I to stage V: pollen mother cells, tetrad, uninucleate microspore, binucleate pollen, and mature pollen stage) were selected for observation by DIC (Fig. 4a-e) and inflorescence microscopies (Fig. 4f-j). The development process can be described as follows. The pollen development starts with the division and differentiation of germline cells into pollen mother cells (PMC) (Fig. 4a, f). Following the meiosis, PMC generates four haploid microspores (Fig. 4b, g) that develop into mononuclear microspore (Fig. 4c, h). The mononuclear microspore gives rise to binucleate pollen after an asymmetric mitotic division (pollen mitosis I, PMI). The binucleate pollen consists of two cells, a larger (vegetative cell) and a smaller (generative cell) (Fig. 4d, i). The second miotic cell division (pollen mitosis II, PMII) of generative cell generates the trinucleate pollen grain. Within the cytoplasm of the bigger vegetative cell, the trinucleate pollen grain contains two smaller sperm cells (Fig. 4e, j). As compared to the male gametophyte of Arabidopsis (Fig. 4k-t), those observations showed that the pollen development process of *Suaeda salsa* is similar to that of the model plant Arabidopsis [25].

### Female gametophyte development of *Suaeda salsa*

**Fig. 5:**
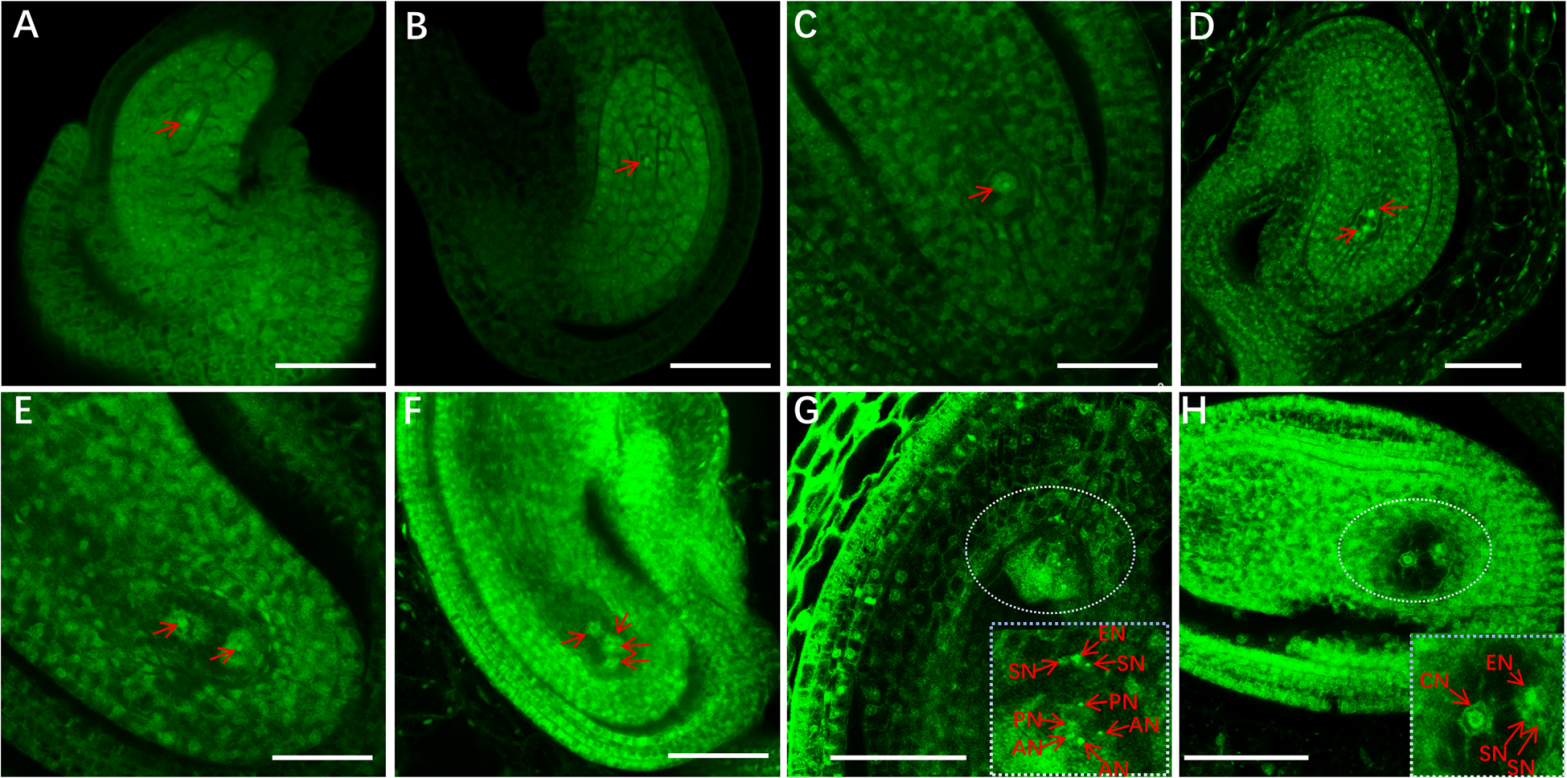
The female gametophyte development of *Suaeda salsa*. **a** Embryo sac with megaspore mother cell, the arrowhead denotes the megaspore mother cell nucleus. **b** Putative meiosis stage. **c** Mono-nuclear embryo sac, FG1 embryo sac, the arrowhead denotes the functional megaspore. **d** Early bi-nuclear embryo sac**,** the arrowhead denotes the nucleus after division, FG2. **e** Later bi-nuclear embryo sac, the arrowhead denotes the nucleus after division, FG3. **f** Tetra-nuclear embryo sac, FG4. **g** Medium eight-nuclear embryo sac. FG5 ~ FG6, the rectangular part in the right corner is an enlarged view of the ellipse part. **h** Later eight-nuclear embryo sac, three antipodal cells have degenerated, FG7, rectangular part in the right corner is an enlarged view of the ellipse part. A-E: Bars = 20 μm. F-G: Bars = 50 μm. AN, Antipodal cell nucleus. PN, Polar nucleus. CN, Central cell nucleus, EN, Egg cell nucleus. SN, Synergid cell nucleus

The female gametophyte is crucial for the sexual reproduction of higher plants [26]. Identification of the developmental type of female gametophyte can promote understanding and carrying out the process of sexual reproduction and hybridization in plants. Taking the developmental process of female gametophyte in Arabidopsis as a control (Additional file 3: Figure S1), we observed the female gametophyte development process of *Suaeda salsa* using WCLSM (Whole-mount-stain clearing laser scanning confocal microscopy) theology. The results showed that the female gametophyte development of *Suaeda salsa* could also be divided into seven stages: Stages I to VII. The development of female gametophyte first begins with megaspore mother cells (MMC, Fig. 5a). At the next stage, compared with the MMC period, the outer integument has completely wrapped the embryo sac, no obvious nuclei can be observed (Fig. 5b). We speculated that this is the meiosis stage, which will give rise to a functional megaspore (FM, Stage I, Fig. 5c). Next, FM undergoes mitosis to form two nuclei (Stage II, Fig. 5d). And soon, the two cells move to each pol, respectively (Stage III, Fig. 5e). Next, the 2 nuclear embryo sac undergo a mitosis to form a 4-nuclear embryo sac (Stage IV, Fig. 5f), and finally the 4-nuclear embryo sac undergoes a mitosis to form a mature embryo sac comprised of seven cells, and 8 nuclei including 2 synergid cells, 1 egg cell, 2 polar nuclei, and 3 anti-pod cells (Stage V~ Stage VI, Fig. 5g). When mature, three antipodal cells degenerate, and two polar nuclei fuse into a central cell (StageVII, Fig. 5h). In the sexual reproduction of angiosperms, two sperms fuse with the egg cell and central cell, respectively. The results showed that the development process of *Suaeda salsa* belongs to the Sputum sac type, similar to that of the model plant Arabidopsis.

### Diploid *Suaeda salsa* has nine pairs of chromosomes

**Fig. 6:**
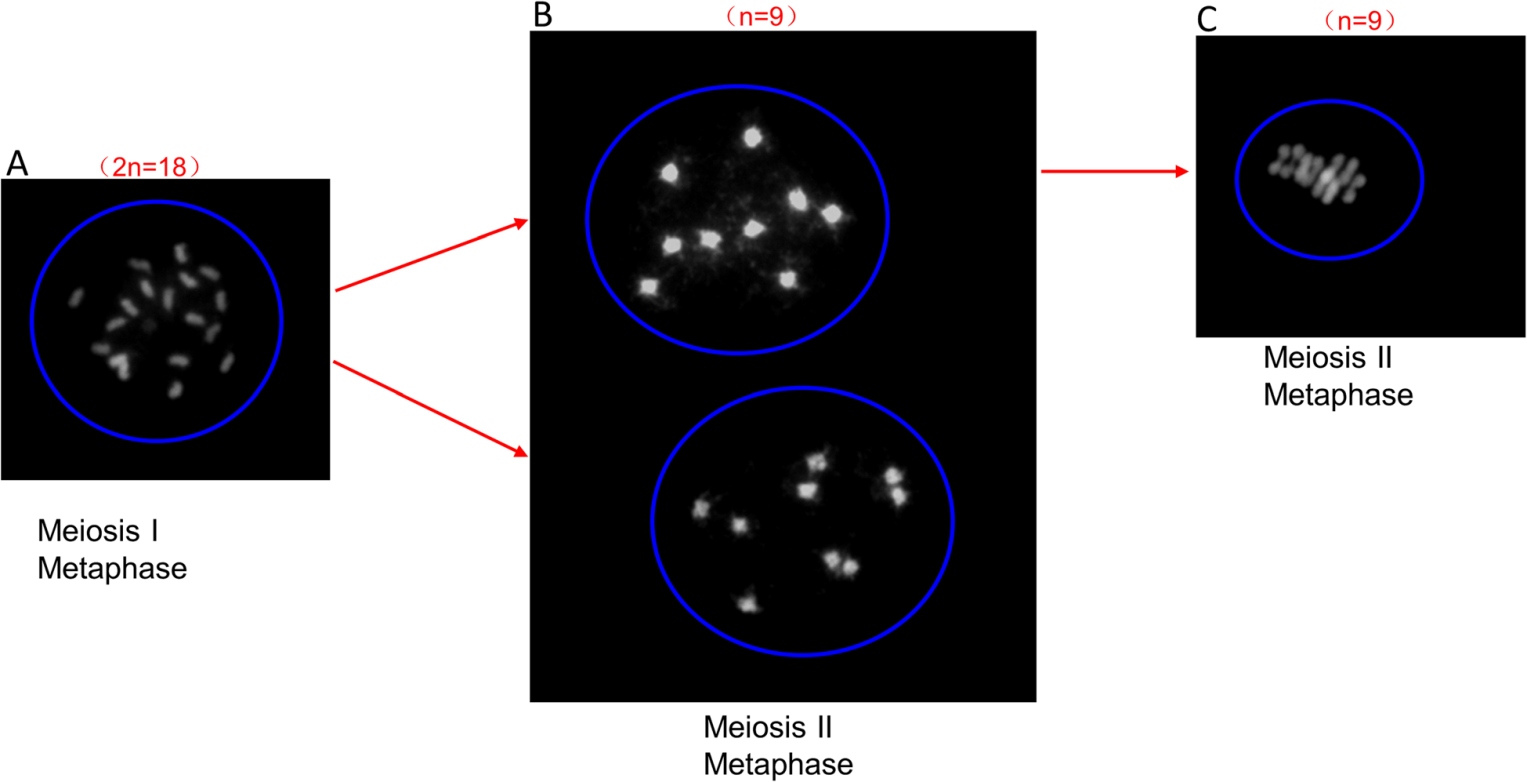
Chromosome behaviour in meiosis of pollen. The pollen was dyed with DAPI and the samples were mounted on the slides for fluorescence microscopy. **a** Meiosis I, Metaphase. **b** Meiosis II, Metaphase of two poles. **c** Meiosis Metaphase of one pole

Studies on chromosomal behaviour and pairing during meiosis of pollen mother cells play an essential role in plant genomic analysis and determination of polyploid types. Observation of chromosomal behaviour during plant meiosis often helps to identify plant ploidy levels [27], In recent years, the chromosome number of many species have been determined by observing the meiosis of pollen mother cells [28]. For *Suaeda salsa*, several reports have given different chromosome counts [29]. To confirm the chromosome number of our *Suaeda salsa* cultivar. The chromosome spreads of microsporophytes were prepared and observed under the microscope. As shown in Fig. 6, we found 18 chromosomes in metaphase of meiosis I (Fig. 6a). Meanwhile, two sets of 9 chromosomes were observed in dyad cells in metaphase of meiosis II (Fig. 6b). Those observations indicated that the diploid *Suaeda salsa* has nine pairs of chromosomes (2n = 18).

### The genome size of *Suaeda salsa* is approximately 437.5 MB

**Fig. 7:**
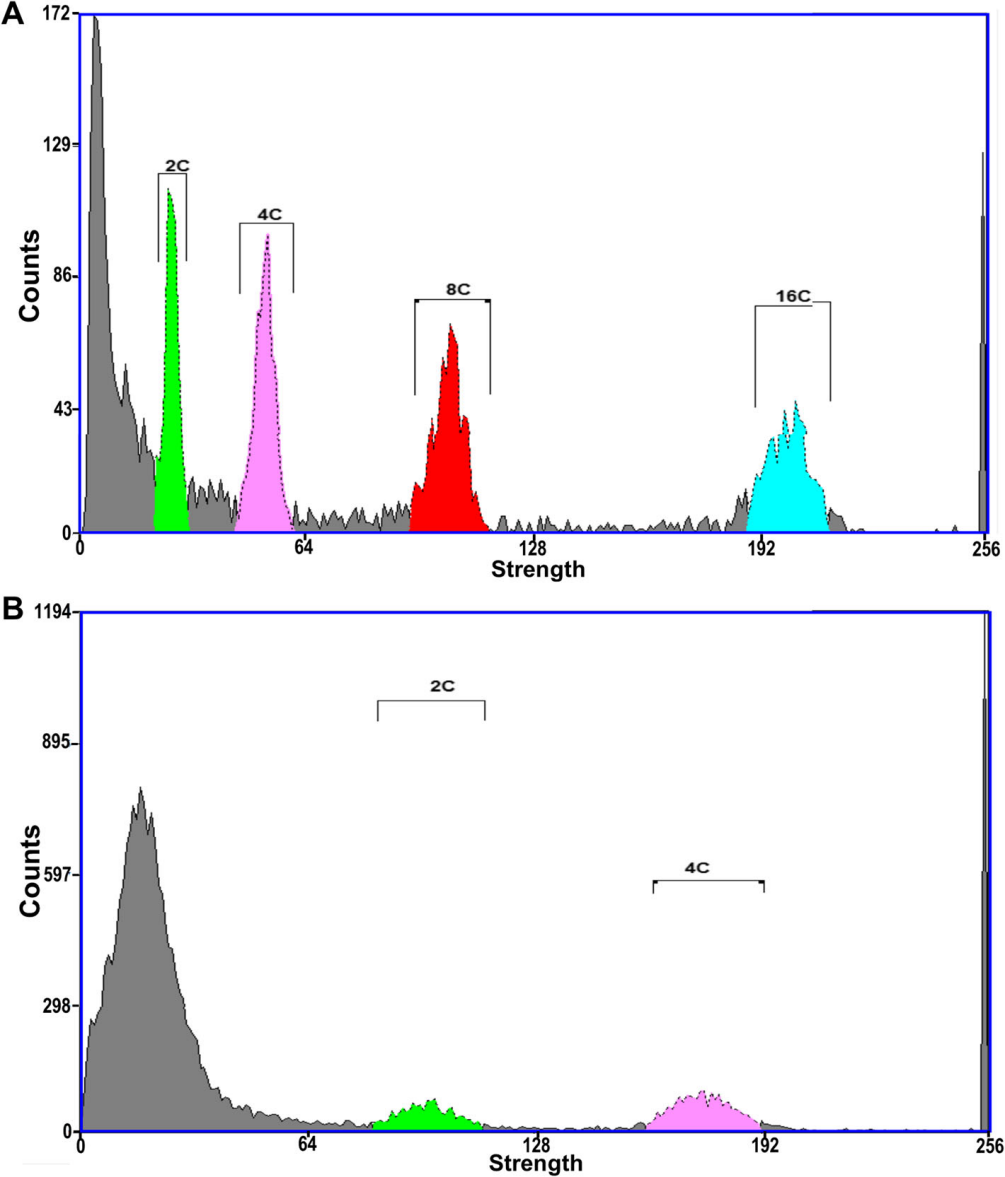
The estimated genome size of *Suaeda salsa* was 437.5 Mb by flow cytometry. **a** The flow cytometry figure of *A. thaliana* leaf showed four peaks: 2C = 24, 4C = 48, 8C = 96, and 16C = 192. **b** The flow cytometry figure showed the 2C and 4C peaks of *Suaeda salsa* leaf, 2C = 84 and 4C = 168. The genome size of *Suaeda salsa* is approximate 437.5 MB

Estimation of the genome size in a species by measuring the DNA content of the nuclei is of considerable significance not only for the molecular, cytogenetic, and genome sequencing of the species but also for the study of plant phylogenetic evolution [30]. The genome size of *Suaeda salsa* was estimated through flow cytometry using Arabidopsis as reference. We first detected 4 peaks (2C, 4C, 8C, and 16C) for Arabidopsis mature leaves with the fluorescence strength of 24, 48, 96, and 192 (Fig. 7a), whilst, two peaks (2C and 4C) with the fluorescence strength of 84 and 168 were detected for leaf nuclei of *Suaeda salsa* under the same parameters (Fig. 7b). The standard deviation of the fluorescence strength of 2C peak is 5.56 (Additional file 4 Figure. S2). Taking the fact that the genome size of Arabidopsis is proximately 125 MB, we estimated that the genome of *Suaeda salsa* is 437.5 ± 28.96 MB.

## Discussion

Salinity and alkalinity are among major stress cues limiting crop growth and productivity. Soil salinization and alkalization have become a global environmental problem that severely affects the sustainability of agriculture. The main reason for the increase in the area of deteriorated land is human activity and climate change [6]. The halophytes are the species withstanding high salt concentrations that kill 99% of other glaucophytes [31]. One type of halophyte, usually dicotyledonous, shows optimal growth at a high NaCl concentration, while the other types of halophytes, generally grow optimally in the absence of salt or at a low NaCl concentration [32]. As the degree of salinization of cultivated land has intensified, researchers are paying more attention to the study of halophytes. According to the statistics, there are about 587 halophytic species in China [33], among which *Suaeda salsa* is the most typical one. The growth of *Suaeda salsa* is significantly stimulated by 200 mM NaCl [8], showing significant salt-tolerance. Additionally, the nutritive value of *Suaeda salsa* mentioned above makes it star species attracting the attention of biologists working on salt tolerance improvement of cultivated crops. Recently, several studies on *Suaeda salsa* have been published focusing on physiology, nutrition, and transcriptome [12, 20, 34]. However, to our knowledge, studies on the fundamental biological characteristics of *Suaeda salsa* are rarely reported until today.

A seed is an embryonic plant enclosed in a protective outer covering [35]. Yielding seeds have been an important development in the sexual reproduction and the success of gymnosperm and angiosperm plants during the evolution, compared to the primitive plants such as ferns, mosses, and liverworts [36]. The seed of *Suaeda salsa* is of typical type with a tough seed coat (Fig. 1a, n). This characteristic is an adaption of *Suaeda salsa* to drought and salinity conditions in the seashore, which could prolong the dormancy and is beneficial for the diffusion prorogation of the species. Seed germination in plants mainly is three types: Hypogeal Germination, Epigeal Germination and, Vivipary (Viviparous Germination). When the *Suaeda salsa* seeds are germinating, the hypocotyl significantly elongates before the emergence of true leaves and brings the cotyledon above the soil (Fig. 1q-s), showing its epigeal germination type. The model plant Arabidopsis, and most other dicots species such as castor, cotton, papaya, onion also belong to epigeal germination type [37].

One characteristic of land plants is the alternation of generations, which is also known as metagenesis. Metagenesis is a type of life cycle that occurs in plants and algae in the Archaeplastida and the Heterokontophyta with distinct sexual haploid and asexual diploid stages [38]. Liverworts, mosses, and hornworts are gametophyte-dominant, while the seedless vascular plants and angiosperms are sporophyte-dominant. *Suaeda salsa* gametophyte is much reduced to the minimum of several cells and relies on sporophyte to obtain nutrition [39]. The haploid female and male gametophytes are essential reproductive units of flowering plants [35]. Male gametophyte development begins with the division of a sporophyte cell. In this study, we described the male gametophyte development of *Suaeda salsa* with five developmental stages, from microspore mother cell to mature pollen containing three cells (Fig. 4). Since two sperms have already formed in the mature pollen grains, the development of male gametophyte of *Suaeda salsa* belongs to the minority type, which is represented in ~ 30% of plants. The mature pollen grain of majority plants (~ 70%, for instance, plants from Solanaceae and Liliaceae) contains only two cells, one vegetative and one generative, and the latter one undergoes the second mitosis just after pollen germination, giving rise to two sperms required for double fertilization [26]. The female gametophyte is a unique structure comprised of quite a few cells and contains the sexual cells [40]. A well-developed female gametophyte is the basis of plant reproduction. Generally, more than 15 different patterns of female gametophyte have been described in angiosperms, which could be classified into three major types: Monosporic (Polygonum) type, Bishopric (Alisma) type, and Tetrasporic (Drusa) type [35]. Although the mature pollen morphology has been reported a decade ago [41], this is the first time to observe and describe the male gametophyte development in *Suaeda salsa*.

The observation of the female gametophyte development of *Suaeda salsa* is challenging. At first, we attempted to reveal its development process through DIC microscopy. However, the experimental results indicated that this method is not quite effective because the complete development process cannot be observed due to the dense embryo sac structure. Partial development stages of female gametophyte development were shown in Additional file 5: Figure S3. We also tried the method of resin section. Unfortunately, we could either not obtain a set of images shown the complete development process of female gametophyte development of *Suadea salsa* (Additional file 6: Figure S4), probably due to the thin oval wall and the limited number of generative cells in one ovule. Eventually, taking advantage of WCLSM (Whole-mount-stain clearing laser scanning confocal microscopy) technology, we successfully observed the development process of the female gametophyte of *Suaeda salsa*. The observations support that the female gametophyte development pattern of *Suaeda salsa* belongs to Polygonum type (Fig. 5). It has been reported that the plants from family of *Brassicaceae* (e.g., Arabidopsis, Capsella, Brassica), *Gramineae* (e.g., maize, rice, wheat), *Malvaceae* (e.g., cotton), *Leguminoseae* (e.g., beans, soybean), and *Solanaceae* (e.g., pepper, tobacco, tomato, potato, petunia) [42–45]) showed Polygonum type female gametophyte development. The observation in this study provides a new example from family *Amaranthaceae*, which also adopts the Polygonum development type of female gametophyte. The healthy development of the male and female gametophytes and the successful completion of the double fertilization directly determine the seed yield. *Suaeda salsa*, as an excellent potential oil crop, scientists nowadays are trying to increase its yield. Here, we revealed the developmental types of male and female gametophytes, which would lay an important foundation for molecular breeding of *Suaeda salsa.*

The chromosome is the vector of genetic information in eukaryotes, it is a combination of Deoxyribonucleic acid (DNA) and protein molecules with part or all of the genetic material of an organism. Typically, the number of chromosomes is constant for all individuals of a specific species, and this is of great importance in determining the phylogeny and taxonomy of the species [46]. Genome is the sum of total genetic material in the haploid set of chromosomes. For most angiosperms, the somatic cell of sporophyte contains two haploid sets of genomes, while the generative cell of gametophyte only has one set. Even though several databases are focusing on the chromosome number and genome size, the extensive observation and investigation on the chromosome number and genome size of specific species are still imperative and of great significance. The chromosome number of *Suaeda salsa* has been reported in the 1950s and 1960s [41, 47]. In the Chromosome Count Database (http://ccdb.tau.ac.il/home/), 16 Chromosome number records of this species (Accepted name: *Suaeda maritima subsp. salsa (L.) Soó*) were deposited with the 2n = 18, 36, 54, respectively (Additional file 2: Table S2) [29]. The inconsistency on chromosome counts records of this species is probably due to the different ecotypes that were investigated. Most of the records have 36 or 54 chromosomes were from Siberia area [48–52]. To confirm this significant characteristic of *Suaeda salsa* of our ecotype, we observed its chromosome behaviour during male gametogenesis. Our results showed that the chromosome number of *Suaeda salsa* is 2n = 18 (Fig. 6). It has been reported that most of the species form *Chenopodiaceae* have relatively stable chromosome organization with the basic number of 9. The exceptions are *Camphorosma* and *Spinaci*a, whose basic chromosome numbers are 6. Our observation is consistent with this conclusion [53]. Taken together, we can speculate that X = 9 might be the original basic chromosome number of *Dianthus* order. Moreover, the estimated genome size of *Suaeda salsa* haploid is approximately 437.5 ± 28.96 MB through flow cytometry in this study (Fig. 7). The results from chromosome number observation and genome size estimation provide useful information for the genomic research on *Suaeda salsa*.

## Conclusions

In this study we observed the seed, plant and floral organ morphology and development of *Suaeda salsa*, the results indicating that the seed germination pattern of *Suaeda salsa* belongs to epigeal germination, and the developments of both male and female gametophytes of *Suaeda salsa* are similar to those of model plant Arabidopsis. The chromosome number of *Suaeda salsa* is 2n = 19. The genome size of *Suaeda salsa* is approximately 437.5 MB estimated by FCM. The observations and results discussed in this article will provide us with a better understanding of the salt (stress)-tolerant plant and insights into future research on *Suaeda salsa*.

## Methods

### Plant materials and growth conditions

*Suaeda salsa* seeds were provided by Yancheng Lvyuan Salt Soil Agricultural Technology Co. Ltd., Yancheng, Jiangsu, Southeast China (http://www.ychpz.com/index.asp). Seeds were treated with 0.03% Gibberellin and planted at 25 °C in the greenhouse with 16/8 h of light-dark photoperiod cycle. The *Suaeda salsa* plants growing in the greenhouse are flowering during summer. The wild-type *Arabidopsis thaliana (L.) Heynh* (Col-0; CS60000) was obtained from the Arabidopsis Biological Resource Center (Columbus, OH, USA; https://abrc.osu.edu/). Arabidopsis plants were grown in a greenhouse with 60% humidity under a 16 h light/8 h dark photoperiod cycle at 22 °C. Flower buds of different developmental stages were used from matured plants for observation of male and female gametophyte development.

### Plant morphology observation and measurement

The photographs showing the plant morphology of *Suaeda salsa* were taken at 10, 20, 40, 80,100 days after germination (DAG). The leaves and lateral stems of *Suaeda salsa* from 100 DAG were dissected and photographed using a Nikon D7200 digital camera. The flower buds at different developmental stages were picked up with a tweezer. The floral organs were dissected with 0.1 mm syringes under an anatomical microscope and then placed on agar plates (0.8%) for photographing. The images were taken through a Leica DFC550 microscope, and the measurements were performed using ImageJ software (NIH).

### Observation of male gametophyte development

Male gametophyte development was observed by both differential interference contrast (DIC) microscopy and inflorescence microscopy. For DIC microscopy, the pollens of different development stages were obtained and cleared by chloral hydrate solution (chloral hydrate: H2O: glycerol = 8: 2: 1) on slides. Cleared anthers were imaged using a BX63 microscope (Olympus) with DIC optics. For inflorescence microscopy, the samples were decoloured in 25% acetic acid 75% ethanol solution for three times and stained with 4′, 6-Diamidino-2-Phenylindole (DAPI), following the method described by Yang et al. (2009) and Dou et al. (2011) [54, 55]. The nuclei of male gametophytes were then observed under Leica MZ10F and DM2500 microscopes.

### Observation of female gametophyte development

The flower buds at different developmental stages were collected and fixed in FAA solution (50% ethanol: glacial acetic acid: formaldehyde =89:6:5) for 24 h. The samples were then washed with 50% ethanol twice and transferred into 70% ethanol for storage. The ovaries were dissected from the fixed florets under a dissecting microscope. WCLSM (Whole-mount-stain clearing laser scanning confocal microscopy) theology [56, 57] was applied to observe the female gametophyte development of *Suaeda salsa*. The dissected ovaries were hydrated sequentially in 50% ethanol, 30% ethanol and distilled water, and mordanted in 2% aluminum potassium sulphate for 20 min followed by staining with eosin (10 mg/L in 4% sucrose solution) for 10–12 h. The stained samples were then retreated with 2% aluminum potassium sulphate for 20 min to remove the dye from the wall of the ovaries. After three times of rinsing with distilled water, the samples were treated successively with 30, 50, 70, 90 and 100% ethanol for 20 min each for dehydration. For cleansing, the dehydrated samples were treated in ethanol-methyl salicylate solution (V: V = 1:1) for 2 h, and then kept in methyl salicylate solution for at least 2 h. The cleansed samples were placed on concavity slides and mounted with fingernail polish and photographed under a Leica SP8 Laser scanning confocal following the reported method [58].

### Chromosome number analysis

The pollen grains with 0.3–0.5 mm in diameter were collected and used for chromosome number observation. The chromosome spreads of microsporophytes were prepared as described previously by [59] and stained with 1.5 μg/ml 4,6-diamidino-2-phenylindole (DAPI). Images of chromosome spreads were taken using a Zeiss (Model) microscope.

### Genome size estimation by FCM

Since *Arabidopsis thaliana* has been sequenced, and its genome size is known (*n* = 125 MB, The Arabidopsis genome initiative, 2000) [60], it was selected as reference in this analysis. The fresh leaves of tested species were dissected from the plants and chopped by a very sharp razor blade in 1 ml Arumuganathan and Earle Buffer [61]. The suspension was then filtered through a 30 μm mesh and 1:100 DAPI (10 mg/ml) was added for nuclei staining. The sample was left for at least 5 min before being analyzed using MoFlo XDP Sorter (Beckman). Typically, 4000–5000 nuclei were measured in each run. The inflorescence strength of different peaks of the Arabidopsis and *Suaeda salsa* were recorded by the instruments and the C- values for different peaks of DNA histogram were generated by the Beckman software. The inflorescence strength of the 2C peak was used for estimating the genome size of *Suaeda salsa* [62].

## Additional files.

**Additional file 1: Table S1.** The size of each organ at five developmental stages.

**Additional file 2: Table S2.** The chromosome number records of Suaeda salsa (L.) Pall. exported from Chromosome Counts Database (CCDB).

**Additional file 3: Figure S1.** Female gametophyte development of *Arabidopsis* under the DIC field.

**Additional file 4: Figure S2.** The flow cytometry assay of *Suaeda salsa*. The figure showed the standard deviation of 2C peak.

**Additional file 5: Figure S3.** Female gametophyte development of *Suaeda salsa* under the DIC field.

**Additional file 6: Figure S4.** Ovary section of *Suaeda salsa*s from early to late stage.

## Data Availability

All data and material are provided in the manuscript and Additional Files.

## Abbreviations

FCM: Flow cytometry
MB: Mega Base pair
WCLSM: Whole-mount-stain clearing laser scanning confocal microscopy
