# Supplementary material for: Studies on genome size estimation, chromosome number, gametophyte development and plant morphology of salt-tolerant halophyte Suaeda salsa
Source: BMC Plant Biol. 2019 Nov 6;19:473. doi: 10.1186/s12870-019-2080-8 (PMC6833229; doi:10.1186/s12870-019-2080-8)
Supplement: Supplementary file 1 — Additional file 1: Table S1. The size of each organ at five developmental stages. [file 12870_2019_2080_MOESM1_ESM.pdf]

Additional Files

Additional Table 1. The size of each organ at five developmental stages.

**Floral Parts**	**Dimensions (mm)**	**Stage1**	**Stage 2**	**Stage3**	**Stage4**	**Stage5**
Bud(mm)	Length(mm)	0.3±0.12	0.7±0.17	1.2±0.14	1.8±0.11	2.1±0.13
	Width (mm)	0.34±0.1	0.74±0.17	1.3±0.15	0.17±0.10	2.1±0.15
Anther	Length(mm)	0.2±0.05	0.4±0.03	0.7±0.02	0.9±0.07	1.0±0.10
	Width (mm)	0.1±0.01	0.2±0.03	0.4±0.10	0.4±0.05	0.4±0.11
Ovary	Length(mm)	0.3±0.12	1.7±0.11	1.8±0.17	2.0±0.14	2.1±0.13
	Width (mm)	0.3±0.11	0.3±0.14	0.3±0.12	0.3±0.16	0.3±0.10
Petal	Length(mm)	0.4±0.06	0.4±0.10	0.5±0.06	0.6±0.11	0.9±0.13
	Width (mm)	0.4±0.14	0.3±0.08	0.4±0.09	0.5±0.10	0.9±0.10
Calyx	Length(mm)	0.5±0.10	0.5±0.11	0.4±0.03	0.3±0.07	0.2±0.03
	Width (mm)	0.3±0.04	0.2±0.02	0.2±0.03	0.3±0.01	0.2±0.04

Four individual plants were used for this assay. Three samples for each plant were measured. Deviation indicates the SD of 12 samples
