# Supplementary material for: Studies on genome size estimation, chromosome number, gametophyte development and plant morphology of salt-tolerant halophyte Suaeda salsa
Source: BMC Plant Biol. 2019 Nov 6;19:473. doi: 10.1186/s12870-019-2080-8 (PMC6833229; doi:10.1186/s12870-019-2080-8)
Supplement: Supplementary file 2 — Additional file 2: Table S2. The chromosome number records of Suaeda salsa (L.) Pall. exported from Chromosome Counts Database (CCDB). [file 12870_2019_2080_MOESM2_ESM.pdf]

Additional Table 2. The chromosome number records of *Suaeda salsa (L.) Pall.* exported from Chromosome Counts Database (CCDB).

Name	Accepted Name	Gametophytic (n)	Sporophytic (2n)	Data Source	Reference
Suaeda salsa (L.) Pall.	Suaeda maritima subsp. salsa (L.) Soó		36	IAPT/IOPB	IAPT/IOPB Chromosome Data 18
Suaeda salsa (L.) Pall.	Suaeda maritima subsp. salsa (L.) Soó		36	IAPT/IOPB	IAPT/IOPB Chromosome Data 8
Suaeda salsa (L.) Pall.	Suaeda maritima subsp. salsa (L.) Soó		36	IAPT/IOPB	
Suaeda salsa (L.) Pall.	Suaeda maritima subsp. salsa (L.) Soó		54	IAPT/IOPB	
Suaeda salsa (L.) Pall.	Suaeda maritima subsp. salsa (L.) Soó		18	IPCN online	Hekmat-Shoar, H. & H. Manafi. 1982. In IOPB chromosome number reports LXXV. Taxon 31: 361.
Suaeda salsa (L.) Pall.	Suaeda maritima subsp. salsa (L.) Soó		36	IPCN online	Lomonosova, M. N., A. A. Krasnikov & S. A. Krasnikova. 2001. Chromosome numbers of Chenopodiaceae from Siberia. Bot. Žhurn. (Moscow & Leningrad) 86(9): 145–146.
Suaeda salsa (L.) Pall.	Suaeda maritima subsp. salsa (L.) Soó		36	IPCN online	Lomonosova, M. N., A. A. Krasnikov & S. A. Krasnikova. 2003. Chromosome numbers of Chenopodiaceae family members of the Kazakhstan flora. Bot. Žhurn. (Moscow & Leningrad) 88(2): 134–135.
Suaeda salsa (L.) Pall.	Suaeda maritima subsp. salsa (L.) Soó		36	IPCN online	Freitag, H. 2006. Typification and identity of Suaeda crassifolia, S. prostrata and S. salsa, three often confused species of Suaeda sect. Brezia (Chenopodiaceae, Suaedoideae). Willdenowia 36(Special Issue): 21–36.
Suaeda salsa (L.) Pall.	Suaeda maritima subsp. salsa (L.) Soó		36	IPCN online	Lomonosova. 2006. Chromosome numbers of some Chenopodiaceae representatives of the flora of Russia. Bot. Žhurn. (Moscow & Leningrad) 91(11): 1757–1759.

Suaeda salsa (L.) Pall.	Suaeda maritima subsp. salsa (L.) Soó		36	IPCN online	Lomonosova. 2005. Chromosome numbers of Chenopodiaceae species from Russia and Kazakhstan. Bot. Žhurn. (Moscow & Leningrad) 90(7): 1132–1134.
Suaeda salsa (L.) Pall.	Suaeda maritima subsp. salsa (L.) Soó		36	IPCN online	Lomonosova. 2005. Cytogeography of the genus Suaeda Forssk. ex J. F. Gmelin (Chenopodiaceae) of Euro-Asia. Pages 55--56 in Karyology, Karyosystematics and Molecular Phylogeny. St. Petersburg, Russia.
Suaedaheteroptera Kitag.	Suaeda maritima subsp. salsa (L.) Soó		18	IPCN online	Probatova, N. S., E. G. Rudyka & S. A. Sokolovskaya. 1998. Chromosome numbers in vascular plants from the islands of Peter the Great Bay and Muravyov-Amurskiy Peninsula (Primorsky territory). Bot. Žhurn. (Moscow & Leningrad) 83(5): 125–130.
Suaeda heteroptera Kitag.	Suaeda maritima subsp. salsa (L.) Soó		18	IPCN online	Shatalova, S. A. 2000. Chromosome numbers in vascular plants of the Primorsky territory. Bot. Žhurn. (Moscow & Leningrad) 85(1): 152–156.
Suaeda heteroptera Kitag.	Suaeda maritima subsp. salsa (L.) Soó		18	IPCN online	Probatova. 2006. Chromosome numbers of plants of the Primorsky Territory, the Amur River basin and Magadan region. Bot. Žhurn. (Moscow & Leningrad) 91(3): 491–509.
Suaeda heteroptera Kitag.	Suaeda maritima subsp. salsa (L.) Soó		18	IPCN online	Lomonosova. 2005. Chromosome numbers of Chenopodiaceae species from Russia and Kazakhstan. Bot. Žhurn. (Moscow & Leningrad) 90(7): 1132–1134.
Suaeda heteroptera Kitag.	Suaeda maritima subsp. salsa (L.) Soó		18	IPCN online	Lomonosova. 2005. Cytogeography of the genus Suaeda Forssk. ex J. F. Gmelin (Chenopodiaceae) of Euro-Asia. Pages 55--56 in Karyology, Karyosystematics and Molecular Phylogeny. St. Petersburg, Russia.

Sixteen chromosome counts records were retrieved from CCDB when searching with the accepted name “Suaeda maritima subsp. salsa (L.) Soó.” IAPT/IOPB: Chromosome numbers published through IAPT Taxon between the years 2006 and 2016; IPCN: Index to Plant Chromosome Numbers including chromosome numbers for plants of all taxonomic groups. The database includes counts originally published in the printed version of the index from 1979 onward. CCDB :Chromosome Counts Database (http://ccdb.tau.ac.il/home/).
