# Supplementary material for: Studies on genome size estimation, chromosome number, gametophyte development and plant morphology of salt-tolerant halophyte Suaeda salsa
Source: BMC Plant Biol. 2019 Nov 6;19:473. doi: 10.1186/s12870-019-2080-8 (PMC6833229; doi:10.1186/s12870-019-2080-8)
Supplement: Supplementary file 3 — Additional file 3: Figure S1. Female gametophyte development of Arabidopsis under the DIC field. [file 12870_2019_2080_MOESM3_ESM.pdf]

Additional Fig. 1

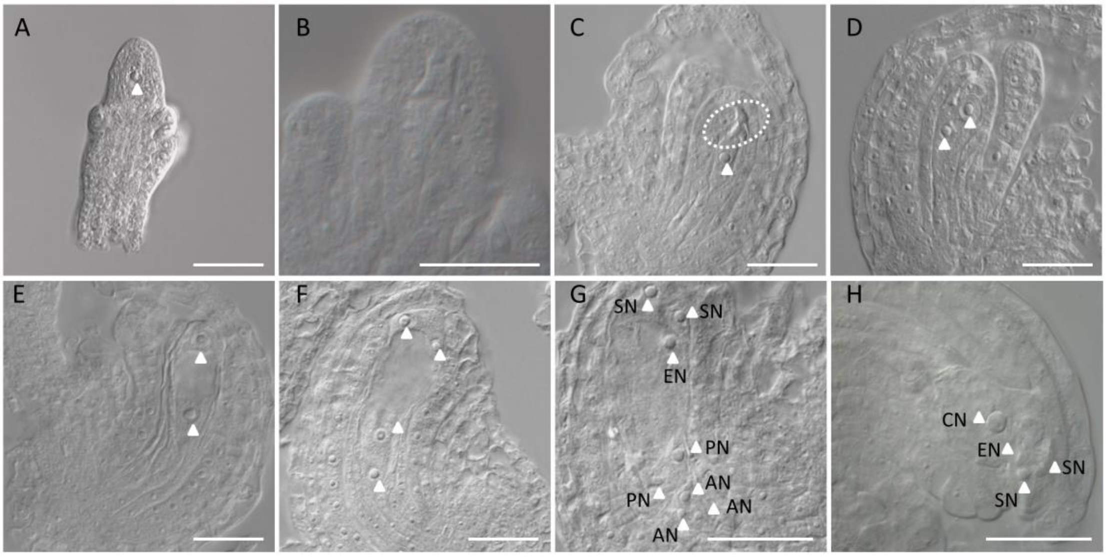

Additional Fig. 1: **Female gametophyte development of *Arabidopsis* under the DIC field.** **(A)** Embryo sac with megaspore mother cell, the arrowhead denotes the megaspore mother cell nucleus. **(B)** The megaspore mother cell undergoes meiosis; at this stage, no apparent generative cell cells could be observed, while the integument could be observed clearly. **(C)** Mono-nuclear embryo sac, the ellipse denotes the degenerative trace of the three megaspores, and the arrowhead denotes the functional megaspore, FG1. **(D)** Bi-nuclear embryo sac, FG2, the arrowhead denotes the nucleus after division. **(E)** Later Bi-nuclear embryo sac, FG3. **(F)** Tetra-nuclear embryo sac, FG4. **(G)** Medium eight-nuclear embryo sac, FG5~FG6. **(H)** Later eight-nuclear embryo sac, FG7. AN Antipodal cell nucleus, PN Polar nucleus, CN Central cell nucleus, EN Egg cell nucleus, SN Synergid cell nucleus. Bars=20μm.
