# Supplementary material for: Studies on genome size estimation, chromosome number, gametophyte development and plant morphology of salt-tolerant halophyte Suaeda salsa
Source: BMC Plant Biol. 2019 Nov 6;19:473. doi: 10.1186/s12870-019-2080-8 (PMC6833229; doi:10.1186/s12870-019-2080-8)
Supplement: Supplementary file 4 — Additional file 4: Figure S2. The flow cytometry assay of Suaeda salsa. The figure showed the standard deviation of 2C peak. [file 12870_2019_2080_MOESM4_ESM.pdf]

Additional Fig. 2

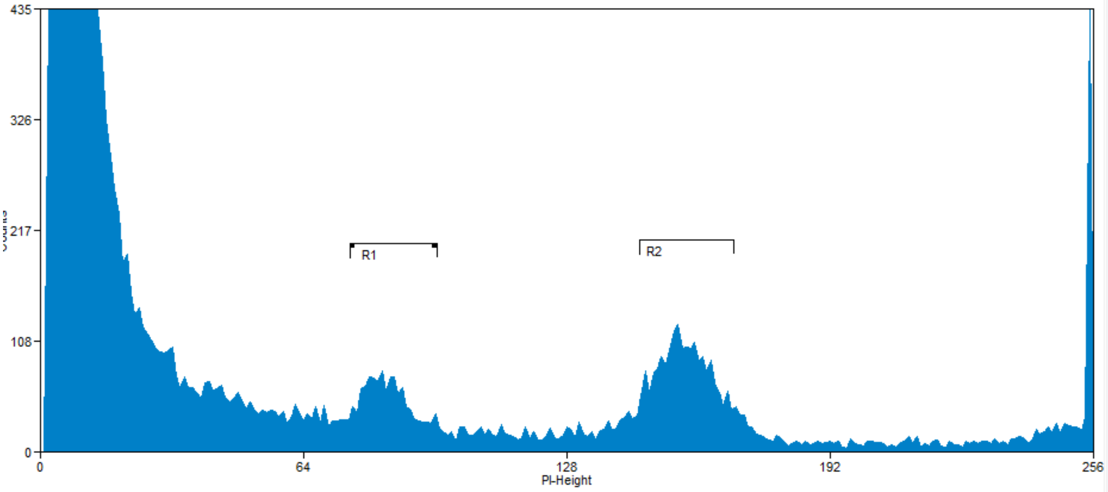

Region	Count	% Hist	% All	Bounds	Mode Count	Mode	Mean	Median	Std Dev.	CV	CV (hm)	Skew
Total	2765...	100.00	100.00	(0.00,255.00)	97381	3.00	10.27	3.00	36.86	359.06	42.37	5.73
R1	1137	0.41	0.41	(75.00,96.00)	79	83.00	84.48	84.00	5.56	6.58	7.15	0.31
R2	1949	0.70	0.70	(145.00,168.00)	125	155.00	156.32	156.00	6.03	3.86	4.65	0.05

Additional Fig. 2. The flow cytometry assay of *Suaeda salsa*. The figure showed the standard deviation of 2C peak.
