# Supplementary material for: Studies on genome size estimation, chromosome number, gametophyte development and plant morphology of salt-tolerant halophyte Suaeda salsa
Source: BMC Plant Biol. 2019 Nov 6;19:473. doi: 10.1186/s12870-019-2080-8 (PMC6833229; doi:10.1186/s12870-019-2080-8)
Supplement: Supplementary file 5 — Additional file 5: Figure S3. Female gametophyte development of Suaeda salsa under the DIC field. [file 12870_2019_2080_MOESM5_ESM.pdf]

Additional Fig. 3

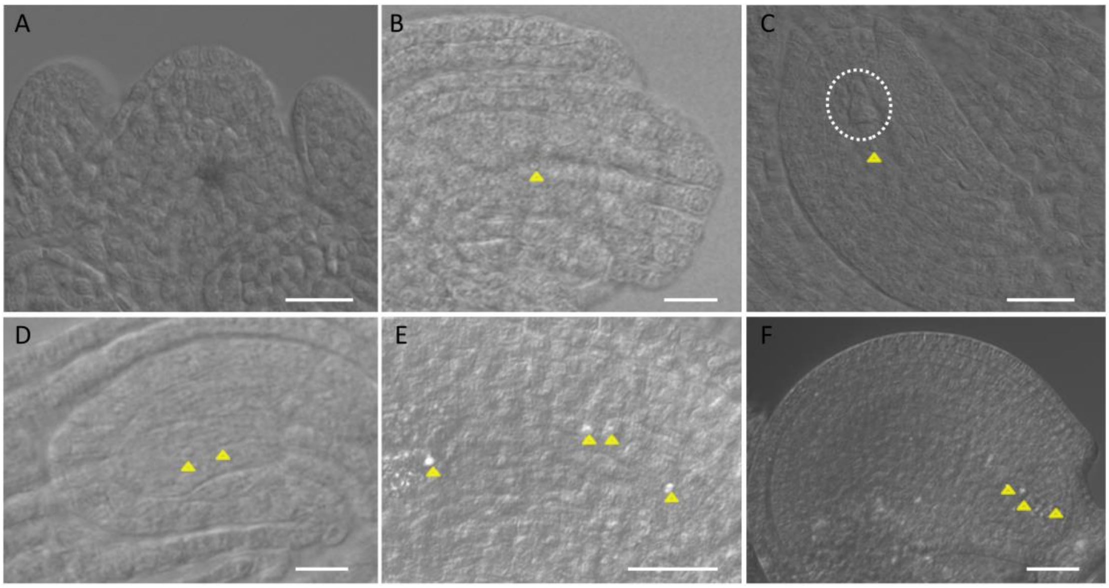

Additional Fig. 3: **Female gametophyte development of *Suaeda salsa* under the DIC field.** **(A)** Early megaspore mother cell stage. **(B)** Megaspore mother cell stage，Figure shown the embryo sac with megaspore mother cell, the arrowhead denotes the megaspore mother cell nucleus. **(C)** Mono-nuclear embryo sac, the ellipse denotes the degenerative trace of the three megaspores, and the arrowhead denotes the functional megaspore, FG1 stage. **(D)** Bi-nuclear embryo sac, FG2 stage, the arrowhead denotes the nucleus after division. **(E)** Putative FG4 stage. **(F)** Later eight-nuclear embryo sac, FG7 stage. Bars=100μm
