# Supplementary material for: Studies on genome size estimation, chromosome number, gametophyte development and plant morphology of salt-tolerant halophyte Suaeda salsa
Source: BMC Plant Biol. 2019 Nov 6;19:473. doi: 10.1186/s12870-019-2080-8 (PMC6833229; doi:10.1186/s12870-019-2080-8)
Supplement: Supplementary file 6 — Additional file 6: Figure S4. Ovary section of Suaeda salsas from early to late stage. [file 12870_2019_2080_MOESM6_ESM.pdf]

Additional Fig. 4

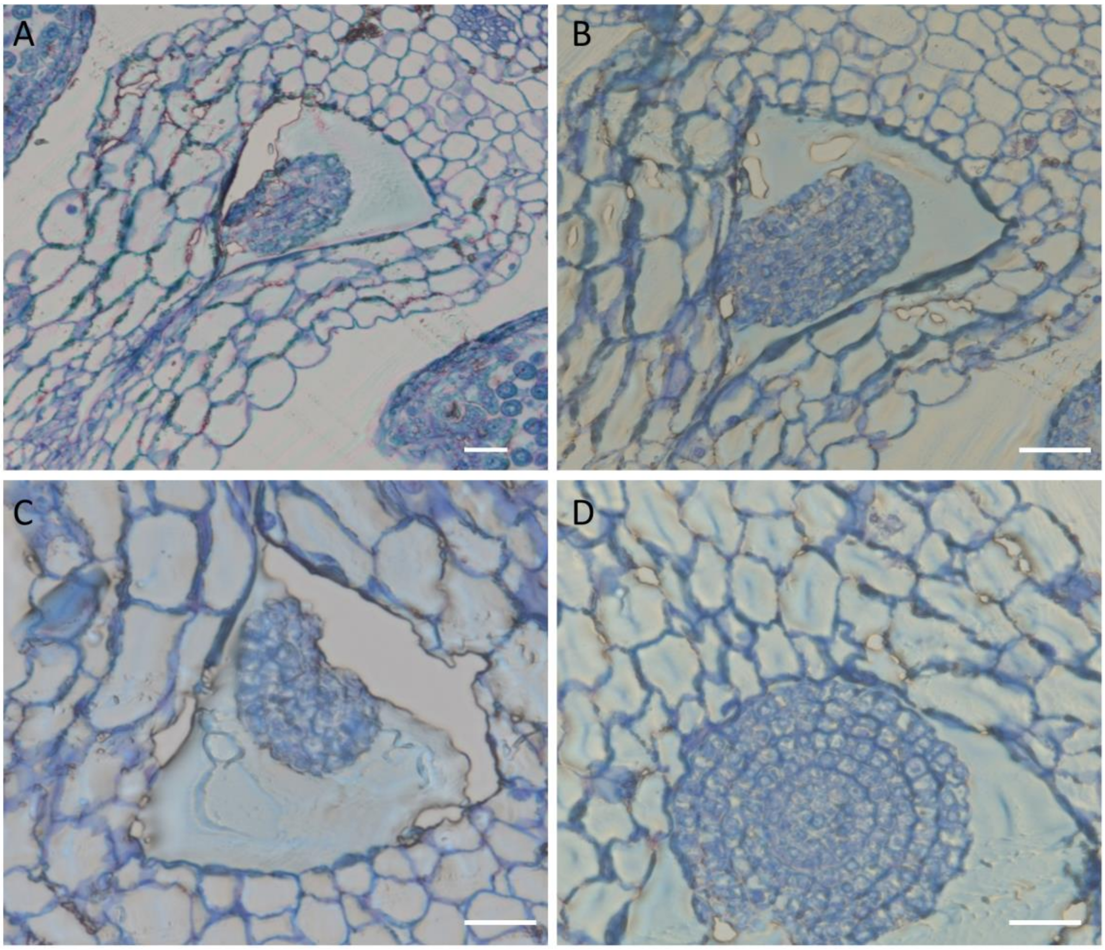

Additional Fig. 4: Ovary section of *Suaeda salsa*s from early to late stage.

(A) Section of the ovary from flower bud during stage I defined by our division. **(B)** Section of the ovary from flower bud during stageIIdefined by our division. **(C)** Section of the ovary from flower bud during stage III defined by our division. **(D)** Section of the ovary after fertilization. Bars=20μm
